# Evaluating the role of clinical officers in providing reproductive health services in Kenya

**DOI:** 10.1186/s12960-018-0296-6

**Published:** 2018-07-11

**Authors:** Marianne Corine Darwinkel, Julius Maina Nduru, Reuben Waswa Nabie, John Anzetse Aswani

**Affiliations:** 1 Community Health Promotion Kenya, P.O. Box 1045, Mtwapa, 80109 Kenya; 2 Kenya Medical Training College, P.O. Box 30195, Nairobi, 00100 Kenya

**Keywords:** Reproductive health, Kenya, Obstetric surgical procedures, Caesarean section, Health manpower, Physician assistant, Specialisation

## Abstract

**Background:**

Most sub-Saharan African countries have too few reproductive health (RH) specialists, resulting in high RH-related mortality and morbidity. In Kenya, task sharing in RH began in 2002, with the training of clinical officer(s)–reproductive health (CORH). Little is known about them and the extent of their role in the health system.

**Methods:**

In 2016, we conducted a retrospective, quantitative two-stage study in Kenya to evaluate the use of CORH and 28 of their curriculum-derived RH competencies, to determine their contribution to expanded access to RH care. CORH were surveyed, using structured questionnaires and telephone interviews. Data on the frequency with which CORH used specified competencies were collected from health records in selected facilities.

**Results:**

Forty-nine of all 104 CORH participated in the survey (47%). Forty-eight (98%) had worked in the clinical area, and 79% were still engaging in clinical work. All 48 worked in emergency obstetrics, emergency gynaecology, and nonemergency RH, and 38 (79%) filled clinical leadership positions. Vasectomy was least performed, by only 9 (18%) CORH. All other competencies were applied by at least half of the CORH, and 22 competencies by more than three quarters. Forty-one (84%) CORH performed caesarean section (CS). Teaching and management were other common responsibilities.

Data were collected from 12 facilities and analysed for 11. They generally confirmed the initial survey findings: CORH worked as obstetrics and gynaecology consultants and used most of their competencies. Analysis was based on 118 months of theatre records. CORH made significant contributions to their facility’s capacity to perform RH surgery: most respondents performed at least 25% of these surgeries. They performed an average of six CS per month and more than 25% of perineal tear repairs (33%), uterus repairs (33%), manual placenta removals (26%), bilateral tubal ligations (39%), and cervical cancer staging (27%). Some experienced CORH conducted procedures beyond their training.

**Conclusions:**

CORH expand access to emergency RH care. Their contributions span all areas of obstetric and gynaecological care, mentoring new health workers and expanding their scope of practice. However, the generally poor status of records documenting healthcare provision limits their usability in evaluation and research.

**Electronic supplementary material:**

The online version of this article (10.1186/s12960-018-0296-6) contains supplementary material, which is available to authorized users.

## Background

Worldwide, 800 women die every day from preventable causes related to pregnancy and childbirth. Almost all of them (99%) live in a developing country [1]. Few countries in sub-Saharan Africa have adequate resources or sufficient skilled health workers to provide critical, evidence-based interventions that improve reproductive health (RH) and reduce maternal mortality [2]*.* Task sharing (or task shifting) is a strategy that countries around the world employ to address gaps in access and availability caused by a shortage of physicians. This strategy creates cadres of associate clinicians^1^ who perform what would traditionally be part of a physician’s job. In 2008, associate clinicians, previously known as non-physician clinicians, were used in 25 out of 47 countries in sub-Saharan Africa [3].

The absence of physicians, especially in rural areas, is problematic when clients need comprehensive emergency obstetric care (CEmOC), specifically caesarean section (CS). Kenya has a very low physician-to-population ratio—approximately five doctors for every 100 000 people [4, 5]—and most of them work in cities and larger towns. Research indicates that associate clinicians, or clinical officers, are present in most health facilities, are less expensive to train and faster to deploy than physicians and achieve similar outcomes. Additionally, there is better retention of clinical officers in rural areas [6–10]. Based on these findings, the World Health Organization (WHO) recommends that clinical officers who have received additional training be allowed to perform CS with ongoing monitoring [2]. In addition to CEmOC, other RH challenges that can be managed by trained clinical officers include management of rape cases, infertility, and early diagnosis and treatment of cervical cancer.

Task sharing with clinical officers has occurred in Kenya for decades; however, information about clinical officers is scarce. For example, the total number of clinical officers in Kenya, their role in the health system, or the extent to which they provide care in rural facilities are not well documented [11–13]. Recent studies suggest that a general lack of understanding of their skills and training causes them to be underused, particularly in health promotion and prevention services [12, 13]. To become a clinical officer, one completes a 3-year post-secondary school training program followed by 1 year internship in a hospital. Completion of this program plus 2 years’ work experience qualifies clinical officers to specialise in one of eight clinical fields: anaesthesia; ophthalmology; ear, nose, and throat; paediatrics; lung and skin; orthopaedics; or oncology. In 2002, the RH specialisation was added to increase the availability of RH services in Kenya, and clinical officer(s)–reproductive health (CORH) have been trained in a one-and-half-year training program followed by a 6-month internship every year since then. The training curriculum was designed to produce skilled associate clinicians who can comprehensively meet the RH needs of individuals, families, and the community, beyond those met by other clinical officers and nurse-midwives. The CORH competencies are managing RH conditions and providing CEmOC, long-term family planning, and medical legal services. Training takes 24 months, six of which are spent gaining practical skills in an internship.

Currently, about 100 CORH work in Kenya’s health system. Despite their formal deployment, CORH do not feature in government policies, regulations, and strategic plans, and literature about them is scant. Kenya has a maternal mortality ratio of 360 deaths per 100 000 births [14]. Considering the ambitious goal of the Kenyan Health Sector Strategic and Investment Plan to reduce maternal and neonatal deaths by half, [15] more information about CORH, the frequency with which they carry out medical procedures, and their role and clinical competencies are essential. Anecdotal information and the few studies indicate that CORH are significantly underused [16, 17].

Our quantitative study evaluated the use of clinical competencies of CORH specified in their curriculum, training manuals, and internship logbooks to assess their contribution to expanded access to CEmOC. The study objectives were to determine the percentage of CORH carrying out emergency and invasive procedures; the frequency with which they carry out specific CEmOC procedures and manage specific gynaecological emergencies; the proportion of surgical procedures performed by CORH relative to other service providers; the diagnostic, family planning, and other non-emergency therapeutic procedures CORH perform; and other specific conditions that they manage in the absence of other skilled healthcare providers.

## Methods

### Study design

This retrospective, quantitative study was carried out in various parts of Kenya in two stages between April and September 2016: a survey among CORH followed by collection of data from health records in various facilities.

The initial survey comprised structured telephone interviews using a questionnaire (Additional file 1). After piloting the questionnaire among general clinical officers and medical officers, 47 interviews with CORH were held between April and September 2016. Interviews focused on the field of the CORH’s work (clinical, teaching, research, or management); the extent to which they used 28 RH competencies in which they were trained (10 in emergency obstetrics, 3 in emergency gynaecology care, and 15 in nonemergency RH care); and in which facilities. These competencies were derived from the CORH curriculum and excluded competencies shared with other cadres with less training, such as normal delivery and provision of non-permanent family planning methods.

During the second stage, data was collected from medical records, using structured data forms. Records came from 12 facilities in 8 out of Kenya’s 47 counties where a CORH worked or had been working. The data-collection form was piloted in a facility not sampled for the study. Inclusion criteria were the following: public facility, in-patient capacity, over 50 deliveries per month, and reported application of most RH competencies by the interviewed CORH. The last criterion aimed at sampling facilities with conducive environments for CORH to carry out their scope of practice, because an earlier study indicated barriers in many facilities [16]. The hospital data analysis therefore indicates the potential contribution of CORH to RH care for Kenyans; it does not represent all facilities where CORH worked.

Data on the frequency with which CORH and other facility staff applied the 28 competencies were collected from the facility in-charges and the facility CORH, general records, labour ward records, theatre books, and, where available, manual vacuum aspiration (MVA) records. We counted the number of procedures performed or assisted by a CORH and the total number of each procedure performed within each facility. Information was collected for 2015, if the CORH was in the facility that year; if not, the nearest year was selected. We found significant problems with record keeping in facilities, making it impossible to collect all desired data from the intended years, resulting in data from nine theatre records, eight labour ward records, and five MVA records.

### Data collectors

Thirteen people were involved in data collection: three investigators and ten research assistants. A 1-day training was conducted for all team members on the tools and telephone interview techniques.

### Recruitment of participants and selection of facilities

Participants were recruited systematically. First, they were identified from a list of all CORH graduates obtained from the training institution. Contact information was then obtained for about half of them from the Clinical Officers’ Council. Using these initial contacts, subsequent contacts were traced through a CORH WhatsApp group. We interviewed all CORH whom we managed to contact and who agreed to be interviewed. Facilities were purposefully selected to include various regions of Kenya and various levels of health facilities.

### Approvals for the study

The study obtained ethical approval from Kenyatta National Hospital and the University of Nairobi Ethics & Research Committee (P645/10/2015), and the National Commission on Science, Technology and Information (NACOSTI) approved the research activities (NACOSTI/P/16/72466/10271). Permission to access records in health facilities was obtained from the Ministry of Health. Kenya’s current devolved county governance system required another layer of approvals from the research department in each county where data were collected.

### Study analysis

All quantitative data were entered directly in Epidata and later exported to Excel for analysis of frequencies.

## Results

### Survey results

#### General respondent information

Data from the Kenya Medical Training College, where the CORH training is conducted, indicated that 104 CORH, 61 (59%) males and 43 (41%) females, had graduated by 2015.^2^ We were able to contact 73 of them for the survey and interviewed 49 (47%). Six CORH declined to be interviewed; 18 agreed to be interviewed but were subsequently unavailable. Twenty-nine CORH could not be reached using their telephone numbers, and two had died.

The 49 CORH interviewed were representatives from all trainings since 2002, except the 2011 training, which had only four students. The distribution of our sample reflects the distribution of the total population of CORH over the various classes.

#### Career paths

Out of the 49 CORH surveyed, 35 (71%) worked primarily in the clinical area, with teaching and management as other common responsibilities. Fifteen CORH (31%) had multiple workplaces, and four (8%) reported carrying out specific clinical CORH competencies at a secondary place of work. Only one CORH never worked in the clinical area, but had always been involved in RH management, teaching, research, and fundraising. All other 48 CORH had managed cases in all the three RH fields: emergency obstetrics, emergency gynaecology, and nonemergency RH. All but 10 (21%) of these 48 CORH had filled clinical leadership positions in RH, by being responsible for one or more of the maternity wards (antenatal, labour, and postnatal), the high-risk antenatal clinics, or the gynaecological and/or gender-based violence clinic. Thirteen CORH (26%) reported responsibilities in all six areas.

#### Application of specific competencies

The survey data showed that, out of the 28 competencies, only vasectomy was rarely performed (Table 1). The reason given was generally “lack of clients.” Out of the 41 who performed CS, 6 CORH commented that they were allowed only to assist with CS or to do it under supervision. Resistance from other practitioners and shortcomings in physical infrastructure and equipment were given as reasons for not performing CS. The three gynaecological emergency care competencies were applied by more than three quarters of the CORH. Three of the CORH (6%) had applied all 15 nonemergency RH competencies, and 11 of these competencies were applied by more than 75% of the CORH. Table 1 contains details on the specified competencies and the percentage of CORH applying them.

**Table 1:** Reported application of specific competencies by 49 clinical officers–reproductive health

Competency	Frequency	Percentage (%)
Emergency Obstetric Care (10 competencies)		
Manage pre-eclampsia*	47	95.9
Manage postpartum haemorrhage	47	95.9
Manage puerperal sepsis	47	95.9
Manual removal of retained placenta	46	93.9
Induce and augment labour	45	91.8
Repair cervical tear	42	85.7
Perform caesarean section	41	83.7
Repair perineum 3rd and 4th degree tear	38	77.6
Secondary repair of burst abdomen after caesarean section	34	69.4
Vacuum extraction of baby	28	57.1
Gynaecological emergency care (3 competencies)		
Perform manual vacuum aspiration	48	98.0
Manage gender-based violence	42	85.7
Manage ectopic pregnancy, including laparotomy	37	75.5
Nonemergency reproductive health (15 competencies)		
Infertility^a^	47	95.9
Visual inspection with acetic acid and visual inspection with Lugol’s iodine	46	93.9
Adolescent reproductive health-related issues^b^	46	93.9
Gynaecological disorders in puberty	44	89.8
Sexual dysfunction	44	89.8
Removal of lost intrauterine contraceptive device	43	87.8
Dilatation and curettage	40	81.6
Bilateral tubal ligation^c^	40	81.6
Insertion and removal of MacDonald stitch	39	79.6
Papanicolaou test	38	77.6
Gestational trophoblastic disease	37	75.5
Staging of cervical cancer	36	73.5
Cervical biopsy	35	71.4
Endometrial biopsy	25	51.0
Vasectomy**	9	18.3

*1 missing value

**9 missing values

^a^ For patients dealing with infertility, CORH are trained in evaluation and counselling of couples, identification and interpretation of laboratory and imaging investigations, and, to some extent, management of infertility, e.g. hydrotubation and myomectomy.

^b^ In light of Kenya’s high teen pregnancy rate, many government guidelines require RH services that are accessible and acceptable to the youth. Competencies of the CORH that related to youth sexual and RH care are diagnosis and management of various menstrual problems and recognition of congenital conditions, including hereditary conditions and problems such as imperforate hymen.

^c^ Only bilateral tubal ligation and vasectomy were included as CORH skills, because the other family planning competencies, such as intrauterine contraceptive device insertion and provision of injectable contraceptives and implants, are also done by other staff (e.g. general clinical officers and midwives).

The survey uncovered additional competencies that the respondents considered part of their practice: management of patients with HIV/AIDS (mentioned thrice), cryotherapy, balloon tamponade,^3^ subtotal hysterectomy, pelvic abscess removal, court attendance in rape cases, leading ward rounds, medical camps, and outreach services (all mentioned once).

### Findings from hospital record data

Data from one of the 12 facilities were not included in the analysis, because few records were available. The 11 remaining facilities varied considerably in RH workload (average deliveries varied from 56 to 687 per month). Two facilities had no operating theatre, and five lacked a gynaecologist.

The survey findings generally were corroborated by the hospital data. General records and information from the facility in-charges and facility CORH confirmed that CORH were mostly used as consultants, in all areas of obstetrics and gynaecology. Survey findings and hospital data both indicated that the participation of CORH in RH surgery varied. In facilities where the CORH had worked many years, he or she appeared regularly in records for elective operations and uncommon emergency procedures and less than expected for common procedures, such as CS. They functioned as de facto consultants and supervisors of medical officers and medical officer interns for common procedures such as CS and MVA. In other facilities, CORH, medical officers, and medical officer interns shared common RH surgery. In a few facilities, tension appeared to exist between medical officers and CORH in allocating the CORH surgical responsibilities. In one facility, this appears to have resulted in a sharp decline in participation by the CORH in theatre accompanied by an observable increase in delivery-related referrals.

Some procedures, although outlined in the curriculum, were never performed by CORH. For example, during the period studied, no vacuum extraction was mentioned in the labour ward records and no vasectomy was recorded in the theatre records. For two procedures, findings from the hospital data were different from the survey results. Examination under anaesthesia for cervical cancer staging was commonly recorded as being performed by CORH, but fewer than 75% of the CORH reported doing so. In contrast, survey data showed that 94% of the CORH engaged in manual removal of the placenta, but this procedure was rarely mentioned in the hospital records.

In none of the 10 facilities with a theatre was the CORH consistently the only RH surgeon. Four of the facilities had relatively numerous staff who performed RH surgery; in six facilities, the CORH worked on a team of two to five RH surgeons. In two of the facilities, the CORH supervised medical officer interns and newly posted medical officers. Overall, CORH contributed much to RH surgery, with most CORH performing at least 25% of them in their facilities.

From the nine theatre records, we analysed data for a total of 118 months (Table 2). The number of CS that the CORH performed each month varied from zero to 31, with a mean of six per month. Most months that the CORH did not perform CS were because they were on leave or out for training. Having a CORH on staff improved CS availability; CORH performed more than half of the CS in almost 40% of the months. Theatre records showed that CORH performed more than 25% of three other obstetric emergency procedures in those facilities (Table 3).

**Table 2:** Caesarean sections per month by clinical officers–reproductive health in nine facilities over 118 months

No of CS per month	Number of months	Percent (%)
No CS	15	16.0
1–4 CS (max. 1/week)	38	40.4
5–8 CS (max. 2/week)	20	21.3
More than 9 CS	21	22.3
	94*	100

*13 months with missing data on CS by CORH and 11 months where CORH refused to go to theatre

**Table 3:** Other emergency obstetric procedures done during 118 months in nine facilities

Procedure	Total	By CORH	
		Number	Percentage (%)
Repair perineal tear*	12	4	33.3
Repair ruptured uterus	9	3	33.3
Manual removal of the placenta	31	8	25.8
Repair cervical tear	40	8	20.0
Re-laparotomy after CS (“burst abdomen”)	30	3	10.0

*Note: Perineal repairs in theatre only reported in two facilities; in the others, no perineal tear repair was reported. This is unlikely and might suggest either grade III/IV perineal tears were often overlooked, or repairs were done in labour ward

We collected data on 641 complicated deliveries from 8 labour ward records. However, analysis of the contribution of the CORH in managing complications during delivery was only possible in one facility. In all other facilities, it was hampered by inconsistencies in, and incompleteness of, the data, including torn and tattered records, many blanks, and failure to record consultations during delivery.

The hospital records showed, on average, between one and two laparotomies for suspected ectopic pregnancy every month. Some theatre records did not record any, whereas the largest facility had 2 months with nine laparotomies for ectopic pregnancy. In the 118 months, 183 laparotomies for ectopic pregnancies were performed, 28 (15%) of these by a CORH.

Only five facilities kept MVA records; even those were scanty. Only one facility kept records in accordance with government guidelines. The five facilities reported between 4 and 47 (a mean of 15) MVAs per month. Only three facilities recorded details about the MVA (indication, who performed the procedure, complications, or referral). The CORH performed 24% of the MVAs in those facilities.

Ten of the 11 hospitals did not have a gender-based violence clinic, but called upon the CORH or a medical officer when a rape case or sexual assault case was reported. It was not possible to get quantitative data that specified the involvement of the CORH.

In the facilities, theatre records were used to assess the frequency with which CORH performed various procedures (Table 4). It was not possible to get substantive quantitative information on nonsurgical consultations by the CORH from the hospital records. In addition to the curriculum-derived competencies, two procedures were clearly within the scope of CORH and frequently performed: marsupialization of Bartholin cyst and avulsion of cervical polyps. No vasectomy was documented. Hydatiform mole, as an indication for MVA, was mentioned once.

**Table 4:** Theatre procedures in nonemergency reproductive health care over the 118 months in nine facilities

Procedure	Overall per month			By CORH per month			Percentage by CORH (%)
	Min	Max	Mean	Min	Max	Mean	
Avulsion of cervical polyp	0	4	0.10	0	1	0.04	40.0
Bilateral tubal ligation	0	13	1.92	0	8	0.75	39.1
Staging of cervical cancer—examination under anaesthesia	0	10	1.34	0	5	0.36	26.9.
Dilatation and curettage	0	11	1.44	0	4	0.35	24.3
Inserting MacDonald stitch	0	9	1.06	0	5	0.25	23.6
Marsupialization of Bartholin cyst	0	6	0.69	0	3	0.16	23.2
Removal of lost intrauterine contraceptive device	0	3	0.13	0	1	0.01	7.7

In line with the survey results, the theatre books displayed records of several procedures performed by CORH beyond the scope of their training curriculum (Table 5). These advanced procedures were mainly carried out by experienced CORH.

**Table 5:** Advanced procedures by clinical officers–reproductive health over the 118 months in nine facilities

Procedure	Number of facilities where the procedures were performed by CORH	Number of procedures performed
Hysterectomy (both subtotal and total)	6	17
Myomectomy	3	4
Ovarian cystectomy	2	3
Incision haematocolpos	2	2
Vulvectomy	1	1
Fistulectomy	1	1
Intra-abdominal abscess	1	1
Prostatectomy	1	5
Herniorrhaphy	1	1

## Discussion

This study initially aimed at assessing the impact of CORH in improving access to CEmOC in Kenya since the inception of the course in 2002. However, in almost all hospitals, records were incomplete and they sometimes contained conflicting data, making inaccuracies in source records a major limitation of this study. Due to these widespread deficiencies, the scope of the study was scaled down to focus on establishing the contribution of CORH in expanding access to CEmOC, gynaecological emergency care, and nonemergency RH care. Where possible, findings were triangulated, e.g. through comparing labour ward records and theatre records or through extracting data from patient files.

All CORH surveyed were still working in Kenya in RH, more than 7 years (on average) after their training. Most CORH provided clinical RH care and many held leadership positions in the clinical field. They applied most of the 28 specialist competencies in their curriculum, with only vasectomy being rarely performed. In addition, 83% of the CORH reported performing major RH surgery, such as CS, a relevant finding as it suggests that more CORH are overcoming earlier reported barriers to carrying out invasive procedures [16]. This retention and integration of associate clinicians in the country and in their field of expertise, in addition to being cheaper to train and deploy, is in line with findings of other studies [7–9] and significant, considering the brain drain to developed countries that Kenya experiences.

Analysis of theatre records clearly demonstrates the significant role the CORH play in saving mothers with complications during delivery by being able to task share in some critical CEmOC procedures, specifically CS and managing post-partum haemorrhage [18]. The contribution of CORH in emergency gynaecological care was less pronounced, relatively smaller, and difficult to assess. Laparotomy for ectopic pregnancy was not performed frequently by CORH. In most facilities, MVA records were poorly if at all kept, making it impossible to ascertain the actual contribution of CORH in performing this simple but lifesaving procedure. From the limited available records, CORH did relatively few MVAs. When these findings were discussed with the participating CORH, some said they mainly engaged in MVAs when supervising juniors (general clinical officers) or when complications arose. It was not possible to determine the role of CORH in non-surgical consultations like for sexual violence or other cases in the obstetric and gynaecological clinic, as the hospital records did not record specific patients or specific cases against specific clinicians.

Some discrepancies were observed between the survey data and the hospital records. A procedure like manual placenta removal was cited as commonly performed by CORH in the survey, but labour ward and theatre records captured very few. In contrast, theatre records showed more frequent staging of cervical cancer by CORH than did the survey results. This finding warrants more investigations as cervical cancer is a significant public health problem in Kenya [19].

Another finding was that some experienced CORH did more procedures than those in the curriculum. For example, both the survey and records indicated that CORH perform advanced surgical procedures such as subtotal hysterectomy, ovarian cystectomy, and draining of pelvic abscesses. This observation could be viewed from two perspectives: CORH could be seen as overstepping the mandate outlined in their curriculum, or they could be seen as a resource to be built on, through official training and task sharing, to make surgical RH care more accessible to communities in remote areas.

In our earlier study [16], the discrepancy in financial compensation between the CORH and other surgical RH staff was problematic. The Kenyan salary and remuneration guidelines state that, for the same work, medical officers get on-call allowance, but CORH do not. Although CORH continued to perform within and even beyond their scope of practice, the discrepancy in remuneration could hinder expansion of CORH services. There were clear indications of this, as two CORH working in the included facilities felt they were misused by the system and had refused to continue working in theatre.

A substantial proportion of the CORH was involved, part-time or fulltime, in training in general clinical officer students. Others mentored other less-experienced health workers—including medical interns and medical officers. Medical officers spend only 3 months in RH during their 1 year internship. Once qualified, they generally work for only one or a few years before moving on to specialise. In comparison, CORH have a more extensive clinical exposure during training (6 months as interns, in addition to 3 months during their basic training) and a more sustained presence at their workstations, making them reliable resource persons in RH for other medical workers. This is significant given that the objective of the initiators of the CORH training was not only to improve access to quality RH care, but also to improve the level of RH training of general clinical officers, by creating role models and trainers in the profession [16].

One additional observation that was not the object of our study, but was quite prominent, was the wide variation in the frequency of diagnosis of certain RH problems, for example post-partum blood loss, and performance of specific RH procedures, for example examination under anaesthesia for cervical cancer or laparotomy for ectopic pregnancy. In several cases, the frequency of diagnosis and procedures varied so widely that it appeared unlikely to reflect variation in prevalence. Possible other causes of this variation could be problems with recognising certain conditions, lack of appropriate referral, or lack of proficiency among advanced RH staff (surgical staff, including CORH) to perform certain procedures.

## Conclusions

Training of CORH is effective in directly increasing human resources for specialist RH healthcare. CORH in clinical practice have roles and responsibilities in all areas of obstetric and gynaecological care, with an emphasis on acute care, specifically CEmOC, and their absolute and relative contributions are substantive. They are retained well within Kenya and within their field of specialisation.

CORH also contribute to RH specialist care through support they give to others in the field, specifically by training and mentoring new health workers, including medical officers and medical officer interns.

Our study is, to our knowledge, the first that has quantified the contribution of the CORH to RH care provision in Kenya. We are only aware of two earlier studies that focused on CORH—our qualitative study and one other study on their involvement in provision of bilateral tubal ligation [16, 17]. The current study has also provided more insight in the role that associate clinicians play in healthcare provision in low-income countries. Rather than relying on existing records, a prospective study with measures of quality control could further assess the impact of task sharing in RH on health outcomes. Despite the costs of such a study, this should be considered as a better understanding of human resources for health has the potential to contribute significantly to better healthcare and better health.

## Additional file

Additional file 1: Clinical Officers Reproductive Health Questionnaire. (PDF 175 kb)

## Abbreviations

CEmOC: Comprehensive emergency obstetric care
CORH: Clinical officer(s)–reproductive health
CS: Caesarean section(s)
MVA: Manual vacuum aspiration
RH: Reproductive health

## Footnotes

1 Associate clinicians and advanced-level associate clinicians include assistant medical officers, clinical officers, clinical associates, medical *licentiate*s, surgical technicians, physician assistants, and advanced-practice nurses. See *World Health Organization* (2012). WHO recommendations: Optimizing health worker roles to improve access to key maternal and newborn health interventions through task shifting, Geneva, Switzerland, 2012. http://www.optimizemnh.org/Annexes/Annex_1_Cadre_definitions.pdf

2
*Information from KMTC – CORH students per batch since 2002 intake*

3 To manage post-partum haemorrhage
