# Supplementary material for: Evaluating the role of clinical officers in providing reproductive health services in Kenya
Source: Hum Resour Health. 2018 Jul 11;16:31. doi: 10.1186/s12960-018-0296-6 (PMC6042229; doi:10.1186/s12960-018-0296-6)
Supplement: Supplementary file 1 — Clinical Officers Reproductive Health Questionnaire. (PDF 175 kb) [file 12960_2018_296_MOESM1_ESM.pdf]

Clinical Officers Reproductive Health (CORH) in Kenya: Evaluation of their utilization and impact on comprehensive emergency obstetric care

Clinical Officers Reproductive Health Questionnaire

IN CASE OF USE IN TELEPHONE INTERVIEW	
Code:	
Date of Interview:	Interviewer Name:
Time interview Started:	Time interview ended:

Good morning/afternoon. My name is ____________. I got your contacts through KMTC/COC. I am calling you because we are carrying out a study to learn more about the Clinical Officers Reproductive Health, about their careers, how much use is being made of their competencies and about the impact they have on the provision of quality reproductive health services in Kenya.

We are studying the fields in which CORH work – clinical area, teaching, research, management, or otherwise. For those who are working in the clinical area we are looking at the extent in which they are able to apply the competencies acquired during their training as CORH. We are also looking at the facilities where CORH have been employed for some time to see if the attachment of the CORH makes a difference in some outcomes in reproductive health, specifically outcomes related to comprehensive emergency obstetric care.

Would you be willing to participate in a telephone interview to enable us to collect some information from you for this study?

If yes, is the current time convenient for you, or would you want us to call you back at another more convenient time?

We are speaking with as many CORH as we can reach and will examine hospital records in various health facilities where CORH are employed. Your responses will help us a great deal in our learning. If you chose to share any personal or sensitive information, please be ensured that it will be kept in confidence. The interview should take no more than 30 minutes, and you should feel free to interrupt us at any time.

If you could please read the consent forms now. I am happy to answer any further questions. We would appreciate your signature before we conduct the interview.

CORH Utilization and Impact Research Consent Form for those interviewed through telephone

Informant: Family name:__________________ First name(s):_________________________

Date (dd/mm/yyyy)_________________

Text to be read out to the potential informant after the explanation about the research project and before starting the interview:

"Do you agree with the following: I understand the CORH Utilization and Impact research project. I understand that consent to participate in the research means giving consent to have the information I share during the interview and / or in filling the questionnaire to inform the CORH Utilization and Impact research. I have understood all that I have read / has been read out and explained to me. I have been given sufficient information and have had my questions answered satisfactorily.

I understand that my participation in the interview is entirely voluntary.

This consent document together with all study data will be kept at Community Health Promotion, Kenya (CHPK), in a safe and secure place.

Do you agree to participate in the CORH Utilization and Impact research project? "

I confirm that I have read out the above text in this consent form in full to the informant and that the informant thereafter agreed to participate in the CORH Utilization and Impact research project.

Staff administering consent form and the subsequent interview:

Name of staff member: _______ _______________________ Signature of staff : _______________

Date: _________ Time: _______ am/pm

IN CASE OF SELF-ADMINISTERED QUESTIONNAIRE THROUGH EMAIL
Code:
Date of Questionnaire filling:

(Study explained in covering email)

CORH Utilization and Impact Research Consent Form for those filling the questionnaire and returning through email

Informant: Family name:___________________ First name(s):__________________________

Date (dd/mm/yyyy)__________________

I understand the CORH Utilization and Impact research project. I understand that consent to participate in the research means giving consent to have the information I share during the interview and / or in filling the questionnaire to inform the CORH Utilization and Impact research. I have understood all that I have read / has been read out and explained to me. I have been given sufficient information and have had my questions answered satisfactorily.

I understand that my participation in the interview is entirely voluntary.

This document will be kept at Community Health Promotion, Kenya (CHPK), in a safe and secure place.

I agree to participate in the CORH Utilization and Impact research project.

Signature:____________________________________ Date:____________Time_________am/pm

A: PLACES WHERE YOU WORKED

1. When did you start your training as CORH? Year: _________ Month: __________

2. When did you graduate as a CORH? Year: _________ Month: ___________

3. What is your main current work station? Name: ________________________________

   Type of facility / organization: ___________
   ____

4. What is your position/title? _____________________________________

5. Tick, how you would rate your main job responsibilities in your current work station (max 2)
   - Clinical □
   - Teaching □
   - Research □
   - Management □
   - Other, to know: __________ □

6. When did you start working here in your current position? Year: _________ Month: ___________

7. Outline which other positions you have had and when in your current work station, if any.
   ____
   ____
   ____

8. Do you have any other work station in addition to your main work station? No □ Yes □

   If, yes, name of the additional work station:
   ____
   Type of facility / organization: ___________
   ____

9. When did you start working in the additional work station? Year: ________ Month: ___________

10. Tick how you would rate your main job responsibilities in your additional work station.
    - Clinical □
    - Teaching □
    - Research □
    - Management □
    - Other, to know: __________ □

11. Did you work in any other places than those already mentioned since you graduated as CORH? No □ Yes □

12. If yes to 11, list the name and type of facilities, the period you worked there, and whether your work responsibilities were mainly clinical, teaching, research, management, or other.

**From (year/month)**	**Until (year/month)**	**Name**	**Type**	**Main job responsibilities**

B: USE OF YOUR MAIN COMPETENCIES, RESPONSIBILITIES AND SKILLS AS CORH
Below follows a list of core competencies, responsibilities and skills of CORH.

For each of them you are requested to indicate if you used them “NEVER”, “ONCE”, “INCIDENTALLY”, “SOMETIMES”, or “REGULARLY” **in the facility where you used this specific competency most.** You can state the frequency as:

- INCIDENTALLY if you normally used it less than one per three months
- SOMETIMES if you normally used it more often than once per three months, but not more than once per two weeks
- REGULARLY if you used it normally more than once per two weeks

If you tick any other than “NEVER”, also indicate the name of the facility, from the ones mentioned in part A, where you used that competency most, and in the last column any other facility where you used that competency

No	Responsibilities	YES / NO	Facility where longest / most responsible / done	Facility where also responsible / done
1	Responsible for antenatal ward			
2	Responsible for labour ward			
3	Responsible for post-natal ward			
4	Run high risk antenatal clinic			
5	Run gynaecological outpatient clinic			
6	Responsible for sexual based violence clinic			
7	Performance obs/gyn surgery in major theatre			
8	Performance obs/gyn surgery in minor theatre			
**No**	**Competencies & Skills**	**Frequency**	**Facility where used most**	**Facilities where used also**
1	To diagnose and manage RH conditions			
2	To provide comprehensive emergency obstetric care			
3	To provide long term family planning services			
4	Provide reproductive medical legal services			
5	Perform obstetrical and gynaecological investigations			
6	Perform obstetric and gynaecological diagnostic and therapeutic procedures.			
7	Request and interpret laboratory and radiological investigations in RH (e.g. infertility)			
8	Manage sexual based violence (rape cases)			
9	Court attendance for sexual based violence cases			
10	Insertion and removal of MacDonald stitch			
11	Perform MVA			
12	Perform D&C			
13	Manage ectopic pregnancy including laparotomy			
14	Vacuum extraction of baby			

15	Removal of retained placenta manually			
16	Removal of retained placenta under general anaesthesia			
17	Repair cervical tear			
18	Repair perineum 3rd and 4th degree tear			
19	Perform Caesarean Section			
20	Secondary repair of burst abdomen following CS			
21	Manage (pre-)eclampsia			
22	Manage postpartum haemorrhage			
23	Manage puerperal sepsis			
24	Induce and augment labour			
25	Perform VILI			
26	Perform BTL			
27	Perform vasectomy			
28	Removal of lost IUCD			
29	Perform VIA			
30	Perform PAP-smear			
31	Perform cervical biopsy			
32	Perform staging of cervical cancer			
33	Perform endometrial biopsy			
34	Perform suction & functional curettage for endometrial cancer			
35	Manage sexual dysfunction			
36	Manage sexuality related issues in adolescence			
37	Manage infertility clients			
38	Manage gynaecological disorders in puberty			
39	Manage gestational trophoblastic diseases including provision of cytotoxic drugs			

C: FACILITIES WHERE YOU USED YOUR CORE CORH COMPETENCIES & SKILLS

We would like to get a bit more information about the facilities you mentioned where you were able to use your core CORH competencies and skills (in the table above).

For the facilities you mentioned, what is their size (bed capacity – approximate), do they have an operating theatre, and which other qualified staff (not interns) were there with basic RH skills (nurse-midwives, midwives, general clinical officers) and advanced/specialist RH skills (other CORH, medical officers, gynaecologists).

No	Name facility	Size (bed capacity)	Operating theatre (YES/NO)	Staff with basic RH skills	Staff with advanced RH skills
1					
2					
3					
4					

Thank you very much for your time. [Recap any information that was especially helpful]

Would you mind if we follow up in the next few months if we have any further questions or need clarification?

You can always contact us through this telephone number or the following email. …… Please do not hesitate to contact us if you have any other questions.
